# Assessment of Dynamic Cerebral Autoregulation in Patients with Basilar Artery Stenosis

**DOI:** 10.1371/journal.pone.0077802

**Published:** 2013-10-04

**Authors:** Xiping Gong, Jia Liu, Pei Dong, Pandeng Zhang, Na Li, Xingquan Zhao, Yongjun Wang

**Affiliations:** 1 Department of Neurology, Beijing Tiantan Hospital, affiliated to Capital Medical University, Beijing, China; 2 Shenzhen Institutes of Advanced Technology, Chinese Academy of Sciences, Shenzhen, China; Charité Universitaetsmedizin Berlin, Germany

## Abstract

**Background and Aims:**

Previous studies have shown impaired cerebral autoregulation (CA) in carotid and middle cerebral artery (MCA) stenosis/occlusion. Little is known about CA in patients with basilar artery (BA) stenosis. We therefore investigated dynamic CA patterns in BA stenosis using transfer function analysis (TFA).

**Methods:**

We measured spontaneous oscillations of blood flow velocity (CBFV) in the right posterior cerebral artery (PCA), and left MCA and mean arterial pressure (ABP) continuously in 25 patients with BA stenosis (moderate n=16 with 50-69% occlusion and severe n=9 with ≥70% occlusion) and 22 healthy volunteers in supine position during 6 circles per minute deep breath. Analysis was based on the ‘black-box’ model of transfer function deriving phase and gain in both PCA and MCA.

**Results:**

Though changes of phase shift and gain between the patients and healthy controls were observed in MCA, the differences are however not significant. Phase shift in PCA was significantly decreased in severe stenosis when comparing with healthy controls and moderate stenosis (4.2±34.2° VS 41.1±40.4°, 4.2±34.2° VS 34.2±27.2°, both *p*<0.05), whilst the gain in PCA is increased for moderate BA stenosis and decreased for severe BA stenosis. Furthermore, we found that phase shift were almost abolished in patients with ischemic stroke who developed unfavorable clinical outcome (mRs>2) on the 90 days after stroke onset.

**Conclusion:**

Dynamic CA in PCA reduces in patients with severe BA stenosis and those with ischemic stroke who present poor outcome in 90 days after stroke onset. Phase shift might be a sensitive index prompting impaired CA in posterior circulation.

## Introduction

Transcranial Doppler (TCD) is a safe and non-invasive technique for assessing hemodynamics of cerebral blood flow especially in evaluation of cerebral autoregulation [1,2]. Over recent decades, methods such as thigh cuff deflation [3], Valsalva maneuver [4] were used to measure CBFV in response to fluctuations of ABP. In addition to inducing changes of ABP, Diehl et al. [5] and Zhang et al. [6] reported that spontaneous oscillations of ABP may also provoke fluctuations of CBFV at the same frequency, allowing assessment of dynamic CA on spontaneous hemodynamic signals. More recently, Diehl et al. [7] proposed a method in assessing dynamic CA using transfer function analysis on Mayer waves (0.06-0.12 Hz) of CBFV in PCA and beat-to-beat ABP induced by deep breathing at the frequency of 6 circles per minute (cpm).

Previous studies focused mainly on CA in carotid and middle cerebral artery stenosis/occlusion, e.g. Reinhard et al. found phase shift between ABP and CBFV estimated from the affected MCA in the patients with occlusive carotid artery disease were significantly lower than that in normal subjects, suggesting impaired CA in these patients [8]. Many studies revealed impairment of CA is associated with TIAs and stroke occurrence in patients with carotid stenosis [9,10]. Characteristics of dynamic CA on carotid and middle cerebral artery have been described widely, while the knowledge of dynamic cerebral autoregulation (dCA) on posterior circulation remains scarce. This is of great concern, as high incidence of intracranial artery stenosis in Asian populations and high risk of stroke in posterior circulation [11]. It remains unclear if CA is also impaired in patients with BA stenosis. Haubrich’s study showed that the high-pass filter model of dynamic cerebral autoregulation can be applied to PCA [12]. Up to now, few studies have focused on CA in the posterior circulation and the results are contradictory [12,13]. Haubrich et al. [12] showed that damping effects of cerebral autoregulation in PCA are lower than in MCA territory while Nakagawa et al. [13] considered such impairment is likely the result of metabolic vasodilation and not an inherent difference in the autoregulatory characteristics of the posterior circulation. Posterior circulation seems to be more vulnerable to the changes of ABP when compared to the anterior circulation [14]， and the incidence of stroke in patients with BA stenosis is higher than anterior circulation [11]. Therefore we proposed the current study to investigate dCA in patients with BA stenosis using transfer function analysis (TFA).

## Subjects and Methods

### Subjects

A total number of 25 patients with BA stenosis were consecutively recruited from February 2011 to November 2012 after admission to the Department of Neurology, Beijing Tiantan Hospital. The following inclusion criteria were used for study entry: 1) BA stenosis was identified by TCD with at least 50% occlusion. The diagnostic criteria were according to the Practice Standards for Transcranial Doppler Ultrasound [15]. The BA stenosis in the recruited patients was further confirmed by MRA or CTA. The extent of stenosis was classified according to the Practice Standards for Transcranial Doppler Ultrasound. Mean flow velocities within 80-119 cm/s indicate a moderate degree (50-69%) of BA stenosis; velocities more than 119 cm/s indicate a high degree (≥70%) stenosis. 2) There are no or mild degree stenosis (less than 50%) in other extracranial and intracranial arteries. Therefore, the changed velocities and dCA in PCA were mainly associated with BA stenosis. 3) There are sufficient bilateral temporal bone windows for insonation of the left MCA and right PCA. Exclusion criteria include atrial fibrillation, acute myocardial infarction, and diseases that may disturb autoregulation such as Parkinsons disease, orthostatic hypotension and dysautonomia. The patients were excluded if the existence of posterior communicating artery or the right PCA originated from internal carotid artery was identified by CTA or MRA.

The control group consists of 22 age and sex-matched subjects recruited from our outpatients clinic (15 male and 7 female; mean ± std ages= 52±11 years).

Among the 25 patients with BA stenosis, 7 were asymptomatic, 4 experienced a TIA (2 cases with onset 2 weeks to 11 months prior to study, 2 cases with onset 2 weeks prior to study), and 14 patients suffered acute ischemic stroke located in the posterior circulation (9 cases within 1 week, 5 cases within 2 weeks). MRI scans were performed in all 14 stroke patients. The infarct lesion was located in the temporal lobe/occipital lobe/thalamus (n=9), medulla (n=1), pons (n=6), midbrain (n=4), cerebellum (n=1), and one patient suffered a complicated acute anterior infarction. CA assessment was performed by 6.8 days (Median 6 days, interquartile 4-9 days) after stroke onset. The modified Rankin scale was assessed in the 14 stroke patients after 90 days either clinically or by telephone interview. Modified Rankin scale >2 was considered as poor clinical outcome. All subjects or their designated relatives provided written informed consent forms. Our protocol including consent procedure was approved by the ethics committee of Beijing Tiantan Hospital.

### Methods

Subjects avoided caffeine/tea, nicotine, and alcohol for 12 hours before the data recordings, which were performed in a dedicated study room with constant temperature (20°C-24°C) maintained and external interference isolated. Subjects lay supine with their head supported on two pillows and after resting for 10 minutes, baseline BP was recorded on brachial artery using an automatic BP monitor.

### Transcranial Doppler Method

Ultrasound measurements were taken according to standard TCD criteria [16]. All Doppler measurements were continuously and simultaneously monitored by a Multidop X4 TCD machine (DWL, Sipplingen). 2-MHz probes were fixed bilaterally over the temporal bone windows with a head frame. We located the TCD on left MCA (depth 53-56mm) and right PCA (depth 65-67mm). The location was verified by testing the vasoneural coupling, (selective increase of CBFV in PCA of at least 20% in the presence of a light stimulus).

**Figure 1:**
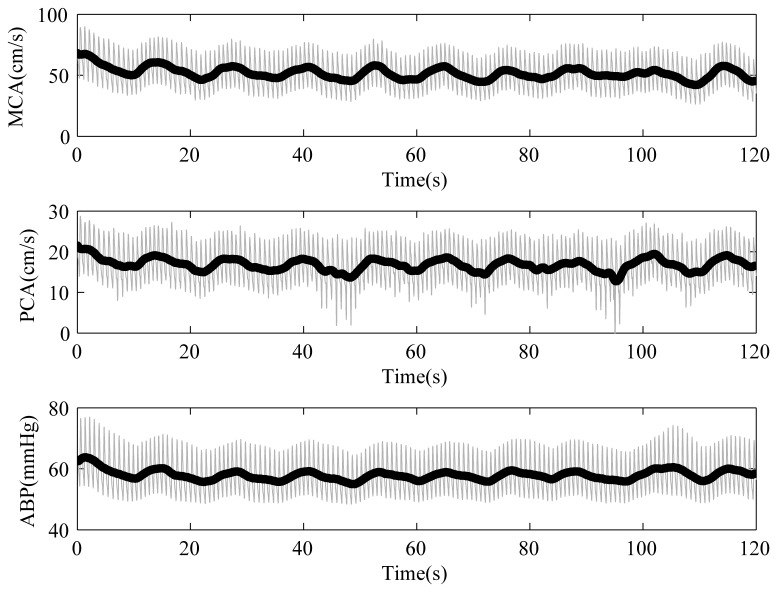
An example of the recorded time series of the finger ABP as well as BFV in MCA (upper plot) and PCA (middle plot) during the paced breathing at 6 cpm. It is evident that the beat-to-beat changes (thick dark line) of these signals are oscillating at the same pace (approximately 0.06-0.12 Hz), which is the frequency of interest for assessing cerebral autoregulation.

All subjects were requested to rest in supine position for 10 minutes before the test. We then started to record CBFV from MCA and PCA as well as ABP simultaneously when patients were breathing spontaneously. After 6 minutes, the patients were guided to breath at the frequency of 6 cpm as shown in Figure 1 minutes and then switched back to normal breathing for another 5 minutes. This was repeated twice for each patient successively.

### ABP Measurement

Continuous beat-to-beat ABP and pulse rate recordings were performed using a reliable servo-controlled finger plethysmograph (Portapres, Ohmeda, U.S.A.), with the subject’s hand maintained at the same level as the heart.

### Data Analysis

Possible time lags were removed by aligning each CBFV and ABP pair using cross-correlation function. A 3rd order Butterworth low-pass filter (cutoff at 0.5 Hz) was then applied as an anti-alias filter before down sampling the data to 1 Hz. The dynamic cerebral autoregulation was evaluated using transfer function analysis [6]. Transfer function between ABP and CBFV was calculated as the quotient of the cross-spectrum of the two signals and the autospectrum of ABP. Impulse and frequency responses were derived from TFA. In frequency domain, we estimated phase, gain, and coherence function within 0.06-0.12 Hz to evaluate cerebral autoregulation, where the derived parameters are most relevant to this hemodynamics [12]. We only used the autoregulatory parameters for the later statistical analysis if the coherence was within 0.06-0.12 Hz >0.5.

The pulsatility index (PI) according to Gosling was calculated as the difference between systolic and diastolic extremes of CBFV divided by the CBFV mean.

Analysis of TCD and ABP recordings were performed offline. Recordings were coded to ensure that data analysis was done blinded to the extent of BA stenosis. All values were given as mean ± std. The difference between groups was determined by Student t test or paired sample t test. Abnormal distributed data were analyzed by χ^2^ test and Mann-whitney test. All data were analyzed using the SPSS 11.5 software package and for each statistical test, *p*<0.05 was taken as significant.

## Results

As shown in Table 1, subjects were divided into 3 groups which are healthy control (n=22), moderate stenosis (n=16, 50-69% occlusion in BA) and severe stenosis (n=9, ≥70% occlusion in BA), respectively. There is no significant difference in CBFV in MCA before and after the paced breathing for all subjects (78.5±15.2 cm/s VS 77.8±14.9 cm/s), suggesting that the maneuver of breath control does not induce hyperventilation.

**Table 1:** Transfer Function Analysis of left MCA and right PCA for all subjects.

	controls (n=22)	Moderate (n=16)	Severe (N=9)	*P* value
Age	50±9	51±11	54±13	0.69
Sex (M/F)	15/7	11/6	6/2	0.87
Vpeak (cm/s)				
MCA	106.2±13.8	110.4±13.1	106.8±15.9	0.63
PCA	47.2±10.5	48.9±13.9	35.7±11.2	0.02*
PI				
MCA	0.83±0.16	0.81±0.16	0.75±0.21	0.48
PCA	0.76±0.17	0.60±0.10	0.46±0.08	<0.01*
Phase shift (degree)				
MCA	36.4±42.7	21.9±44.1	22.6±37.0	0.52
PCA	41.1±40.4	34.2±27.2	4.2±32.2	0.03*
Gain (cm/s/mmHg)				
MCA	1.4±1.2	1.8±1.1	1.8±1.1	0.56
PCA	0.8±0.6	1.5±1.4	0.9±0.4	0.05
Coherence (Index)				
MCA	0.55±0.13	0.60±0.22	0.58±0.20	0.77
PCA	0.52±0.12	0.63±0.16	0.56±0.25	0.16

All values are given as mean ± std, *p<0.05 according to Student’s *t* test.

Abbreviation: MCA, middle cerebral artery; PCA, posterior cerebral artery; PI, pulsatility index; Vpeak, systolic peak velocity.

In Table 1, it shows that CBFV (shown by the systolic peak velocities) or PI in MCA does not change in patients with BA stenosis. However, CBFV in PCA is significantly reduced in patients with severe BA stenosis, which is similar to the situation in PI in PCA, implying normal perfusion in the PCA can possibly be maintained until the occlusion is severe.

**Figure 2:**
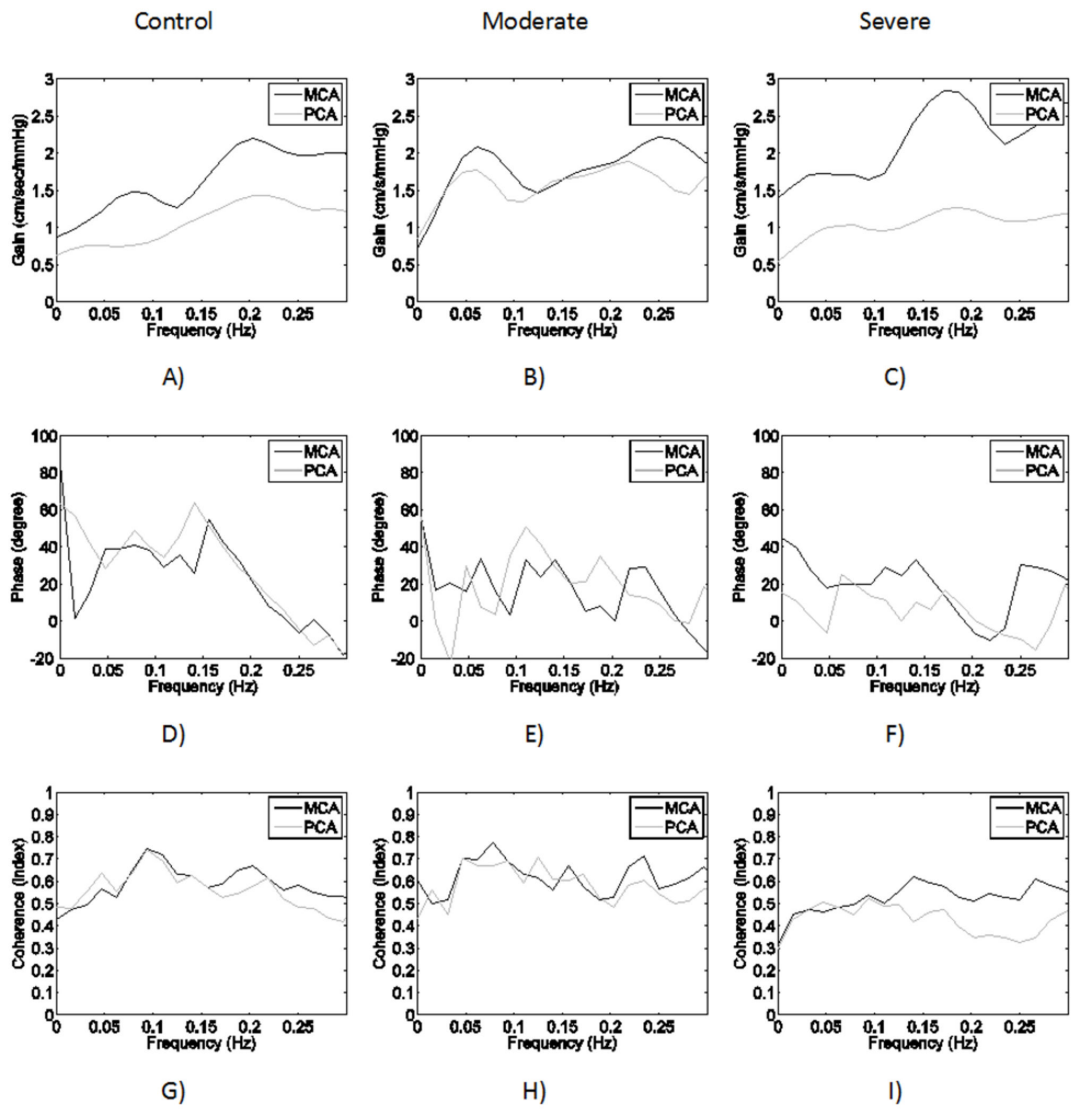
The group-averaged transfer function analysis for the subjects in three categories (control, moderate, and severe) is plotted. The coherence (H-I) between 0.06-0.12 Hz exceeds 0.5 for all conditions, indicating TFA is valid to describe the recorded hemodynamics as linear systems. Control: A) High-pass gains were obtained for both MCA (dark line) and PCA (gray line). D) Large positive phase difference between blood flow velocity and arterial blood pressure in 0.06-0.12 Hz was observed for both MCA and PCA. Moderate: B) The gains are increased in MCA and PCA when comparing with the controls, implying the amplitudes of blood flow velocity follow ABP more passively than the controls. E) The phase in MCA and PCA is reduced. Severe: C) The gains of MCA and PCA move separately. In particular, the gain of PCA becomes flat and low, whereas the gain of MCA is increased. F) Positive phase in MCA is still preserved. However, it is close to zero in PCA, suggesting CA is severely impaired.

The TFA proposed by Zhang et al. [6] was applied to investigate cerebral autoregulation in frequency domain, as plotted in Figure 2 and listed in Table 1. This method is considered valid as the mean coherence (0.06-0.12 Hz) in all conditions exceeds 0.5. Though the stenosis is occurred in BA, cerebral autoregulation might however be affected remotely in MCA as both gain and phase shift for MCA are changed. Figure 2.A-C also confirms this finding. As comparing with the controls, gains are elevated and phase shifts are reduced for MCA (dark line) when BA stenosis is either moderate or severe. However, these changes are not statistically significant.

**Figure 3:**
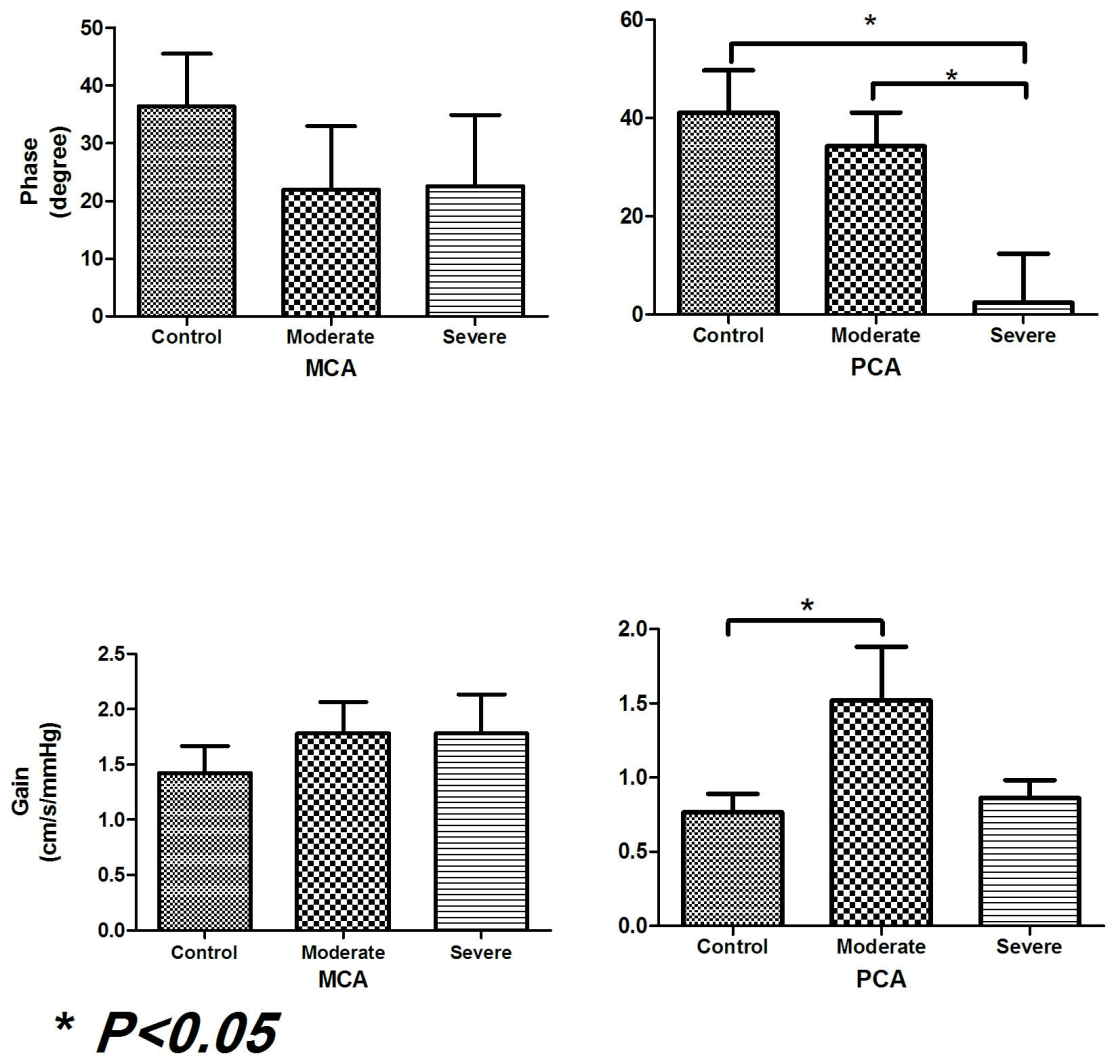
Statistical analysis (ANVOA) of phase shifts and gains in MCA and PCA. For MCA, gains are elevated and phase shifts are reduced when BA stenosis is either moderate or severe. However, these changes are not statistically significant. For PCA, it shows that the reduction of phase shift is proportional to the occlusion, whereas the gain for PCA is increased significantly for moderate stenosis and decreased for severe stenosis in BA.

For PCA, it shows that the reduction of phase shift is proportional to the occlusion (see Table 1), whereas the gain for PCA is increased for moderate stenosis and decreased for severe stenosis in BA (see Figure 3). We think that the decrease of gain for severe stenosis does not necessarily suggest improved autoregulation as CBFV in PCA is significantly reduced (see Table 1) in patients with severe BA stenosis and the shape of the gain, in Figure 2.C (gray line), is flattened (a shape of high-pass gain indicates active autoregulation).

As described above, phase shift appears to be more sensitive to the vessel occlusion in BA. We then attempted to link this parameter to the outcome of stroke patients. Four patients experienced a TIA and 14 patients suffered from acute ischemic stroke located in posterior circulation were enrolled. The phase shifts for PCA were similar in asymptomatic and symptomatic (TIA/stroke) patients (29.5±15.6° VS 16.4±30.3°, *p*>0.05). We found that the phase shift in stroke patients shows descending tendency than those of asymptomatic and TIA patients respectively but without significance (*p*>0.05). All stroke patients received modified Rankin scale assessment on the 90th day after stroke onset. Significant lower phase shifts in PCA were detected in patients with poor outcome (n=6) compared to those without symptom or with slight disability (n=8) (27.8±18.6° VS 4.7±14.6°, *p*<0.05).

## Discussion

This is the first attempt to characterize dCA using TFA for moderate and severe BA stenosis, though impaired dCA was previously identified in patients with severe (≥70%) internal carotid artery stenosis in several studies [8,9,17,18].

The phase shift obtained from respiratory-induced oscillations at 0.1 Hz in ABP and CBFV can be considered as a time lag. A phase shift of 0° indicates total absence of autoregulation, while a large positive phase shift of 40°-70° can be regarded as intact autoregulation. The phase shift represents the response of blood flow to the changes of blood pressure. In our study, the phase shifts obtained from respiratory-induced oscillations at 0.1 Hz in ABP and CBFV for PCA and MCA in healthy subjects were similar, which is consistent with previous studies. Haubrich et al. suggested that phase shift does not significantly differ between both regions of the cerebral circulation [19]. Rosengarten et al. found that cerebral autoregulation in the carotid and vertebrobasilar system does not differ in the time course of the recovery of blood flow velocity following a sudden decrease of ABP using thigh cuff technique on healthy adults [20].

Our study showed that phase shift decreased in PCA in the patients with moderate BA stenosis when comparing with normal condition and the reduction of phase shift in severe BA stenosis is highly significant when comparing with normal and moderate condition. Even 0° of phase shifts were occasionally detected in patients with severe BA stenosis. It suggests that severe BA stenosis has a negative effect on CA in PCA and a strongly passive response of CBFV to ABP could occur in such condition. This agrees with the findings on dCA of MCA in the carotid stenostic circumstance [18].

In clinical conditions, ie, presyncopal symptoms and hypertensive encephalopathy, cerebral autoregulation points towards a higher vulnerability within posterior parts of the brain [21]. In our study no significant difference was found in phase shift in PCA between stroke and nonstroke patients. It may be associated with the stroke pathogenesis in posterior circulation. The stroke lesions were multiple and distributed bilaterally. The mechanism of most ischemic posterior strokes is thought to be of embolic and not of hemodynamic origin.

According to the follow-up result of the 14 stroke patients, phase shifts are significant reduced in patients with poor outcome which implies a deficit of the filter function of CA. The results indicate that the deficit of the filter function of CA in acute stage (within 2 weeks after onset) may contribute to the poor neurologic outcome. This is consistent with other clinical ciucumstances [22-24] including aneurysmal subarachnoid hemorrhage, traumatic brain injury, and large infarction. Study has shown that cerebral autoregulation to small spontaneous fluctuations in ABP being altered in patients with large ischemic strokes, such as malignant middle cerebral artery infarcts [24].

The transfer function gain has been regarded as an indicator of the change of amplitude in CBFV corresponding to the changes in ABP. Generally, a high gain value in low frequency range (0.06-0.12 Hz) indicates a passive CBFV response and is thus considered as impaired autoregulation. In this study, we did not find consistent changes of the gain values in PCA between the patients and controls. In contrast, the phase shifts in PCA are lower (highly significant for severe BA stenosis) in the patients when comparing with the controls. However, the gain in PCA is increased for moderate stenosis and decreased for severe stenosis in BA. It is known that the compensatory dilation of PCA is in consequence to the BA stenosis. Accordingly, the vasomotor capacity of PCA may decline gradually along with the progressing of BA stenosis. This would result in the reduced variation of the diameter and velocity of PCA in response to the fluctuation of ABP. Therefore, we believe that the low gain values in the patients with severe BA stenosis do not suggest improved CA. We thus suggest that transfer function gain values obtained from the relationship between ABP and CBFV flu**c**tuations might be of less significance than that of phase shift.

The limitations of current study are analyzed as follows. Firstly, we only included the patients eligible for transcranial Doppler monitoring, while the patients with poor temporal acoustic bone windows were excluded. This might introduce bias if the excluded samples had significant impact on overall results. Secondly, the samples of our study are relatively small and the subject enrollment lasted more than 20 months due to our strict exclusion criteria for minimizing the impact of other intracranial and extracranial arteries stenosis on hemodynamic patterns. Therefore the conclusions drawn from our study cannot be generalized to the patients with combined stenosis of posterior circulation. Thirdly, we validated BA stenosis by CTA and MRA rather than DSA, as they have been shown as an effective method for detection of stenosis, though DSA is recognized as the ‘gold standard’ of stenositic diagnosis [25].

## Conclusion

Our study showed a lower autoregulatory capacity in the posterior circulation in patients with severe BA stenosis. The phase shift angle in PCA obtained from the relationship between ABP and CBFV fluctuations within 0.06-0.12 Hz might be a more sensitive index prompting impaired CA in BA stenosis.
